# Genome-Scale Identification Method Applied to Find Cryptic Aminoglycoside Resistance Genes in *Pseudomonas aeruginosa*

**DOI:** 10.1371/journal.pone.0006576

**Published:** 2009-11-11

**Authors:** Julie M. Struble, Ryan T. Gill

**Affiliations:** Department of Chemical and Biological Engineering, University of Colorado, Boulder, Colorado, United States of America; University of Missouri-Kansas City, United States of America

## Abstract

**Background:**

The ability of bacteria to rapidly evolve resistance to antibiotics is a critical public health problem. Resistance leads to increased disease severity and death rates, as well as imposes pressure towards the discovery and development of new antibiotic therapies. Improving understanding of the evolution and genetic basis of resistance is a fundamental goal in the field of microbiology.

**Results:**

We have applied a new genomic method, Scalar Analysis of Library Enrichments (SCALEs), to identify genomic regions that, given increased copy number, may lead to aminoglycoside resistance in *Pseudomonas aeruginosa* at the genome scale. We report the result of selections on highly representative genomic libraries for three different aminoglycoside antibiotics (amikacin, gentamicin, and tobramycin). At the genome-scale, we show significant (p<0.05) overlap in genes identified for each aminoglycoside evaluated. Among the genomic segments identified, we confirmed increased resistance associated with an increased copy number of several genomic regions, including the ORF of PA5471, recently implicated in MexXY efflux pump related aminoglycoside resistance, PA4943-PA4946 (encoding a probable GTP-binding protein, a predicted host factor I protein, a δ 2-isopentenylpyrophosphate transferase, and DNA mismatch repair protein mutL), PA0960–PA0963 (encoding hypothetical proteins, a probable cold shock protein, a probable DNA-binding stress protein, and aspartyl-tRNA synthetase), a segment of PA4967 (encoding a topoisomerase IV subunit B), as well as a chimeric clone containing two inserts including the ORFs PA0547 and PA2326 (encoding a probable transcriptional regulator and a probable hypothetical protein, respectively).

**Conclusions:**

The studies reported here demonstrate the application of new a genomic method, SCALEs, which can be used to improve understanding of the evolution of antibiotic resistance in *P. aeruginosa*. In our demonstration studies, we identified a significant number of genomic regions that increased resistance to multiple aminoglycosides. We identified genetic regions that include open reading frames that encode for products from many functional categories, including genes related to O-antigen synthesis, DNA repair, and transcriptional and translational processes.

## Introduction

The objective of this work is to adapt the SCalar Analysis of Library Enrichments (SCALEs) method [1] for use in *Pseudomonas aeruginosa*. The aspiration is that this genomic tool would allow the genome-wide identification of a large number of intrinsic genomic regions and genes related to antibiotic resistance or the evolution of resistance. Specifically, we examined the system of genetic regions related to resistance of *P. aeruginosa* to the aminoglycoside class of antibiotics. This is also the first work in which SCALEs, with modification, has been applied to an organism other than *E. coli*.

Antibiotic resistance is a growing clinical problem [2], [3] resulting in increased disease severity, mortality rates, and healthcare costs. Clinically resistant strains often contain multiple resistance mechanisms, which may contribute to resistance in either an additive or multiplicative fashion [4]–[6]. The genes underlying such mechanisms can evolve via mutation of ancestral genes, including housekeeping genes and metabolic genes [7], [8]. Thus, the process of evolving new resistance phenotypes (aside from the acquisition of new genetic material) depends upon the reservoir of potential resistance genes maintained within a particular bacterium [9]–[11]. Identifying these potential resistance genes can be difficult or laborious using conventional methods such as homology comparison to known resistance genes or genetic tool such as traditional library screening [12] or transposon mutagenesis [13]. As a result, efforts to better predict the evolution of resistance remain challenging [5], [8], [14]–[16].

*P. aeruginosa* is an opportunistic pathogen that is noted for its ability to adapt to a variety of niches and substances that are considered harmful to microorganisms, including antibiotics [17]–[20]. Antibiotics provide pressure for bacteria to acquire or increase resistance mechanisms. The rise of antibiotic resistant organisms is a model of active evolution. Strains able to develop resistance will proliferate while those unable to develop resistance are unable to survive or flourish. Patients that are administered multiple antibiotics or undergo extended antibiotic therapy, such as cystic fibrosis patients, are at higher risk of developing infections with resistant bacteria. *P.* aeruginosa is frequently responsible for severe infections in those affected by cystic fibrosis. Additionally, in hospitals where antibiotics are used extensively, thus providing an environment conducive to evolving resistance, *P. aeruginosa* is frequently isolated (www.cdc.gov).

The aminoglycosides are a class of bactericidal (with a few exceptions) natural or semi-synthetic antibiotics that act by binding to the 30S ribosome with noted effects of inhibition of DNA and protein synthesis, disruption of peptide chain elongation, interference with peptide chain release from ribosomes, and loss of cell membrane integrity [21]–[24]. Resistance to aminoglycosides is well established, often involving aminoglycoside modifying enzymes, ribosomal mutations, altered cell permeability, efflux pumps [25], or ribosomal methylation by 16S rRNA methylase [26]. Nevertheless, the precise mechanisms by which aminoglycosides cause cell death have yet to be fully elucidated.

The SCALEs method involves several steps. The first step involves fragmenting genomic DNA, selecting specific sized genomic fragments, and ligating these fragments into a suitable backbone vector to construct several highly representative (>99% probability that the entire genome is present) plasmid-based genomic libraries of distinct insert size. These genomic libraries are individually transformed into an appropriate host strain, pooled, and subjected to a selective condition to enrich the population for clones best fitted to survive during the selective condition. The plasmid DNA is then extracted from the selected pool of clones and hybridized to a DNA microarray containing probes designed from the genome of the organism from which the genomic libraries were constructed. The multiple size libraries act in a cumulative effect to create signal intensity patterns on the microarray. SCALEs then uses a decomposition of the data which correlates signal intensity of probes on the DNA microarray to genomic position and allows the deciphering of genomic segments enriched within the selected populations [1]. This method goes beyond traditional methods that rely upon conventional sequencing that are limited to the identification of only a handful of the most-fit clones. SCALEs enables the direct assessment of increased dosage effects on cellular phenotypes, which, while not encompassing all possible mutations, has been shown to represent a major source of evolutionary novelty in nature and to mimic many effects (i.e. gene overexpression, altered regulation, etc.) that might also result from point mutations [27].

For this study, we created three highly representational different sized genomic libraries of *P. aeruginosa* PAO1 within the vector pBTB-1 [28] . Vector pBTB-1 is a low copy number broad host range plasmid containing a β-lactamase resistance marker, a pBAD promoter upstream of the cloning site, as well as transcriptional terminators flanking the cloning site to aid in insert stability. These libraries were transformed into the recombination-deficient *P. aeruginosa* PAO1 mutant, PAO2003, pooled, and parallel selections performed to identify via traditional sequencing or SCALEs the genomic regions capable of conferring increased tolerance to amikacin, gentamicin, or tobramycin. These antibiotics are all structurally related but have differing substitution patterns on their aminoglycoside backbones. These differences in structure and substitution patterns impact the activity of each antibiotic [29]. We chose to study three different aminoglycosides in attempts to identify genomic regions capable of conferring resistance to not only a specific aminoglycoside, but also to the more general class of aminoglycosides. Our analysis efforts focused first on validating the reliability of the data provided by the adapted SCALEs methodology and on assessing the enriched resistance regions as well as the overlap among enrichment patterns obtained for each aminoglycoside.

## Results

The overall objective of this work was to adapt the SCALEs methodology to identify at the genome-scale a reservoir of potential aminoglycoside resistance genes present in *P. aeruginosa*. We first created and characterized libraries of mixed insert size (see Methods) and then performed growth selections on mixtures of such libraries. Next, we used conventional sequencing and Affymetrix *P. aeruginosa* Genome arrays to identify genomic regions present in library populations before and after selection. Finally, we assessed overlap among resistance regions enriched in each aminoglycoside selection.

### Growth selections to identify resistant clones

Selections were performed by mixing equal molar concentrations of 1 kb, 2 kb, and 4 kb insert sized genomic libraries within the host strain *P. aeruginosa* PAO2003, allowing those mixtures to recover in non-selective conditions for 1 hour, and then spreading an equal numbers of cells onto plates containing increasing concentrations of gentamicin, tobramycin, or amikacin. All selections resulted in colonies growing on concentrations of antibiotic 1–2 fold greater than the concentration at which the PAO2003 or the PAO2003 strain containing the empty vector backbone were able to grow, indicating that genomic insert contained within surviving library clones were providing a moderate selective advantage. The selections were performed in duplicate, with comparable number of surviving clones achieved each time. The gentamicin library selections had colonies that grew upon concentrations up to 16 µg/ml compared to 4 µg/ml for the controls. Tobramycin library selections had colonies growing up to 4 µg/ml, with a lawn of colonies growing at 2 µg/ml, which was the concentration sufficient to prevent growth of the control samples. Amikacin library selections had clones growing on concentrations of up to 16 µg/ml while the control population was capable of growing on concentrations up to 4 µg/ml. Control selections did have a small number of colonies that grew on antibiotic concentrations higher than the concentration that was necessary to kill the majority of the cells. This raises the possibility that control populations had a small number of mutants or persister clones, which emphasized the necessity of confirming that the resistance of selected library clones was due to increased copy of plasmid-based genomic insert DNA.

### Confirmation and Analysis of Resistance Clones

Ten clones were taken randomly from each selective plate to identify genomic regions contained within plasmid DNA and confirm resistance phenotypes. This also allowed us to assess the diversity among the surviving selected clones and assess the necessity of microarray studies. Here, a clone is defined as a PAO2003 transformant containing a distinct region of genomic DNA within its plasmid based insert DNA. The genomic inserts were identified via sequencing of plasmid DNA isolated from selected clones. Of the 40 clones chosen for further analysis, 31 contained inserts containing overlapping genomic regions. Eight clones were chosen that contained different genomic inserts, their plasmids extracted, and these plasmids transformed back into *P. aeruginosa* PAO2003. Once transformed, these clones were evaluated to confirm resistance phenotypes and assess cross-resistance to other aminoglycosides. Five of the eight distinct clones were found to have increased resistance to the tested aminoglycosides (Table 1). Interestingly, of the three clones that did not exhibit increased resistance, two had multiple inserts and one had an insert of only 62 base pairs. One clone was found that did contain two inserts (A8-C1) and did have confirmed increased resistance. It is not determined whether the increased resistance is due to a combination of the two inserts or whether one of the inserts alone is sufficient.

**Table 1:** Sequenced clones isolated from selections along with their resistance profiles.

Clone/Strain	Start Position^a^	Stop Position^a^	ORFs/genes included	Product
Control: PAO2003	-	-	-	-
Control: PAO2003+pBTB-1	-	-	-	-
T2-C1	5,576,686	5,577,513	PA4967/*parE*	topoisomerase IV subunit B
T2-C9	6,161,493	6,156,835	PA5472	hypothetical protein
			PA5471	hypothetical protein
			PA5470	probable peptide chain release factor
			PA5469	conserved hypothetical protein
			PA5468	probable citrate trasporter
G16-C1	5,547,691	5,551,513	PA4943	probable GTP-binding protein
			PA4944/*hfq*	host factor-I protein Hfq
			PA4945/*miaA*	δ 2-isopentenylpyrophosphate transferase
			PA4946/*mutL*	DNA mismatch repair protein MutL
G16-C2	1,050,226	1,046,460	PA0963/*aspS*	aspartyl-tRNA synthetase
			PA0962	probable DNA-binding stress protein
			PA0961	probable cold-shock protein
			PA0960	hypothetical protein
A8-C1^b^	605,929	2,567,312	PA0547	probable transcriptional regulator
			PA2326	hypothetical protein

a position identified via sequencing.

b clone contains multiple inserts.

To verify whether the increased resistance phenotypes were a factor of the medium used for this study, EZ RDM, or if the medium imposed growth restrictions that influenced the increased resistance, we did two things. We confirmed the increased resistance of the clones T2-C1, G16-C1, and G16-C2 to aminoglycosides in Luria-Bertani broth. We also confirmed that a previously characterized *P. aeruginosa* strain, B1 [30], that has been found to have increased resistance to aminoglycosides in Luria-Bertani broth, maintained the elevated resistance levels in EZ RDM.

After sequencing, we determined that there was genetic variation within the selected populations in that not all sequenced clones contained the same genomic region within their plasmids. Moreover, the 40 colonies examined via sequencing, were a small fraction of the clones surviving the selection, with approximately 1000 to a lawn of colonies remaining on the selection plates. Thus, we expected that gene chip based studies would prove useful at identifying the genomic regions enriched within the large number of clones that were not among the clones isolated for sequencing. We thus harvested the plasmids from the remaining clones and employed the SCALEs methodology to identify the genomic-regions contained within the plasmid-based insert DNA of selected populations.

### Microarray analysis of resistant populations

After each selection, plates containing the clones exhibiting an increase in resistance were scraped and freezer stocks made of the scraped cells. In order to have a sufficient amount of plasmid DNA to detect with a microarray, cells from freezer stock were grown in media containing carbenicillin and glucose to minimize impact of the genomic insert (located behind a glucose-inhibited pBAD promoter) upon growth. Thus, the microarray signal reflects not only the clones that were present after the plate-based selection, but also the growth rate of those clones during the recovery period. As such, the microarray results give an indication of the ability of a particular clone to not only survive at a specific antibiotic concentration but also the overall fitness of that clone in the absence of any antibiotic selective pressure.

**Figure 1:**
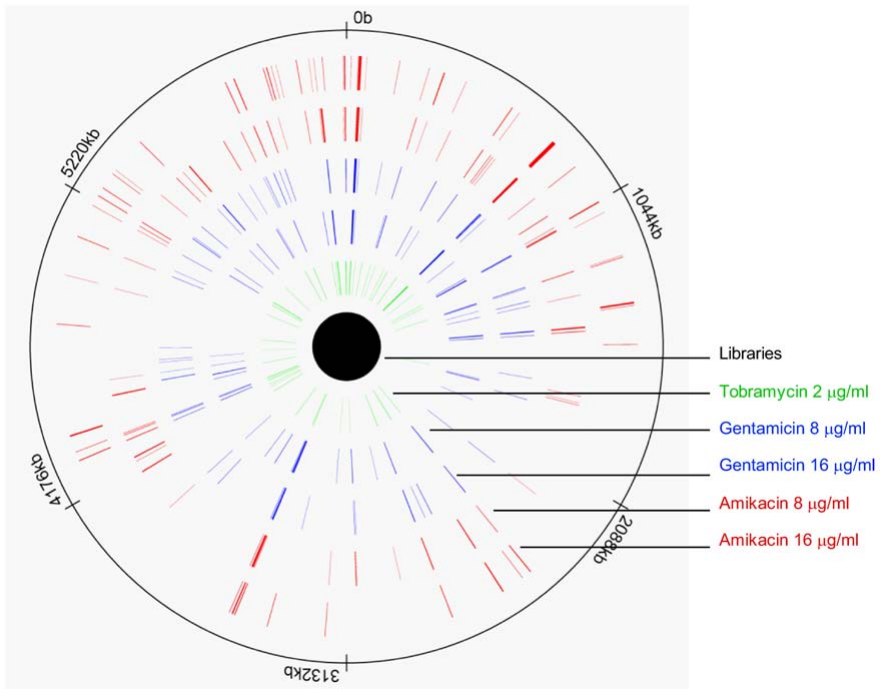
Enriched genomic regions plotted along the *P. aeruginosa* PAO1 genome. The genomic regions highlighted were were enriched during aminoglycoside resistance selections and identified via microarray. The aminoglycoside used in the selection and concentration are given in the labels of the concentric rings. The unselected library sample shows that signal was present all along the genome, indicating proper genomic coverage.

Plasmid DNA within the clones growing upon the plates containing 2 µg/ml tobramycin, 8 and 16 µg/ml gentamicin, as well as 8 and 16 µg/ml amikacin, were separately prepared and hybrized to micoarrays. The genomic regions identified from each selection are depicted as highlighted regions along the *P. aeruginosa* PAO1 genome in Figure 1. Across all selections, similar proportions of particularly sized genomic fragments were enriched. Fragments of 1,500 bp or less constituted roughly two-thirds of the enriched populations. Fragments of greater than 1,500 bp but less than 3,000 bp constituted roughly one quarter of the enriched populations. Fragments larger than 3,000 bp made up for approximately 10% of the enriched populations.

The genomic inserts identified via sequencing of randomly picked clones prior to microarray experiments were among the most prominent regions identified, with the exception of the region of the gene encoding for topoisomerase IV subunit B, which was observed to grow slowly in the freezer stock recovery phase (data not shown). This genomic region is, however, enriched above background noise and can be distinguished if signal intensities greater than 2 standard deviations, rather than our cut-off for dominant peaks of 4 standard deviations, from the mean signal intensity are examined. A list of the common genes and genomic regions found within the dominating peaks of each antibiotic selection is given in Table 2, with a more extensive table of regions identified included in supporting information Table S1. We have examined the homology searches for the ORFs listed as encoding hypothetical proteins, as well as examined the COG (cluster of orthologous groups) [31] predictions and PFAM (Protein Families Database of Alignments and hidden Markov models) [32] predictions of these genes. Of the 17 ORFs listed in Table 1, no predictions could be found for 7 of the ORFs. PA5211 and PA5466 had COG predictions that these genes are related to permease activity and the COG prediction for PA2228 and PA5567 is that of a β-lactamase class C protein and a GTPase, respectively. Of other note is the PFAM prediction of PA4636 as an acetyltransferase.

**Table 2:** Common genes and genomic regions identified in selections.

ORF/gene	Product
PA0041	probable hemagglutinin
PA0727	hypothetical protein from bacteriophage Pf1
PA0985	probable colicin-like toxin
wbpI	probable UDP-N-acetylglucosamine 2-epimerase WbpI
wbpH	probable glycosyltransferase WbpH
wbpG	LPS biosynthesis protein WbpG
wzz	O-antigen chain length regulator
-	intergenic (genomic position 4,292,875-4,297,375)
PA3860	probable AMP-binding enzyme
PA3866	pyocin protein
PA5210	probable secretion pathway ATPase
PA5468	probable citrate transporter
PA5470	probable peptide chain release factor
PA2228, PA2730, PA2852, PA3860, PA4536-PA4537, PA4636, PA5211-PA5212, PA5412, PA5466- PA5467, PA5469, PA5471-PA5473, PA5567	hypothetical proteins

### Overlap among resistant populations

**Figure 2:**
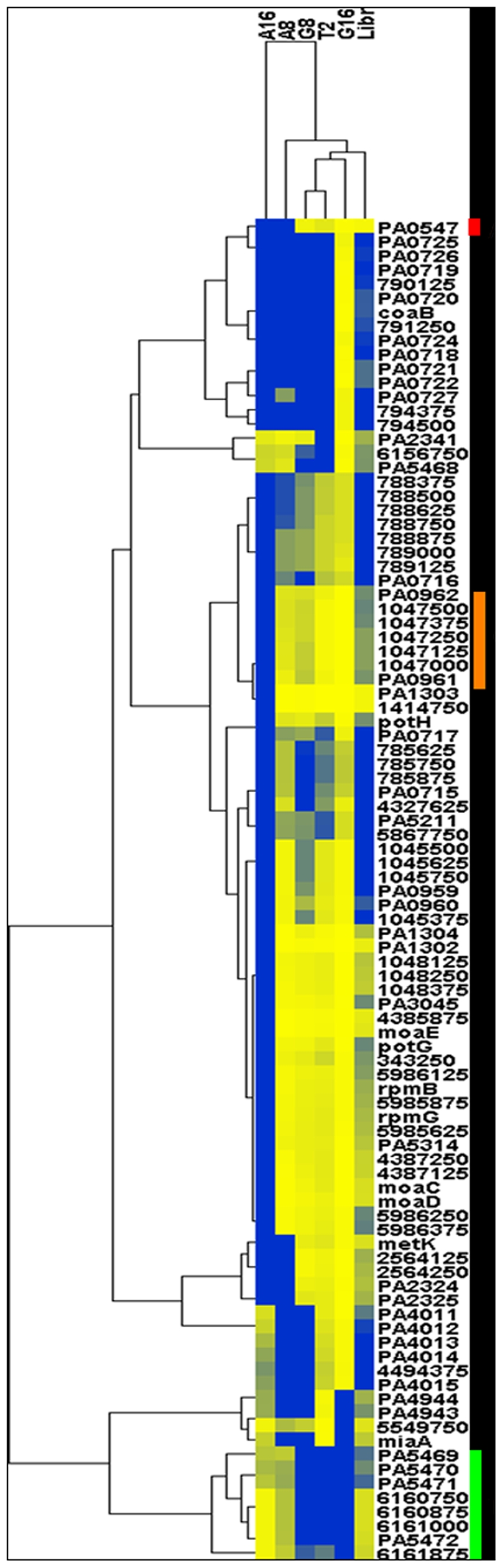
Hierarchical clustering of selection population microarray data, displaying most enriched genetic loci. The selection populations are represented with different columns, with names given across the top. Enriched loci are shown in the rows. Regions colored in yellow are less enriched. Regions colored in blue are more enriched. Loci are listed as genomic position given in the center base pair of the locus, ORF number, or gene name. Colored boxes on the far right correspond to genomic regions identified in conventional sequencing and later confirmed to increase aminoglycoside Resistance. The following abbreviations were used to title arrays: A16 (amikacin 16 µg/ml population), A8 (amikacin 8 µg/ml population), G16 (gentamicin 16 µg/ml population), G8 (gentamicin 8 µg/ml population), T2 (tobramycin 2 µg/ml population), and Libr (unselected mixed libraries population).

The enriched populations overlapped substantially across the different aminoglycoside selections. In fact, pairwise correlation between each of the different enriched populations was significant (p<0.01). To assess overlap among aminoglycoside enrichments, hierarchical clustering was performed to group selections according to genomic enrichment profiles (Figure 2). The population of clones surviving on 8 µg/ml gentamicin and the population of clones surviving at 2 µg/ml tobramycin were found to be the most similarly enriched. The gentamicin selection performed at 16 µg/ml also grouped with these two selections while the amikacin selections were grouped into a distinct cluster. The unselected libraries were the least similar of all of the microarray data.

**Figure 3:**
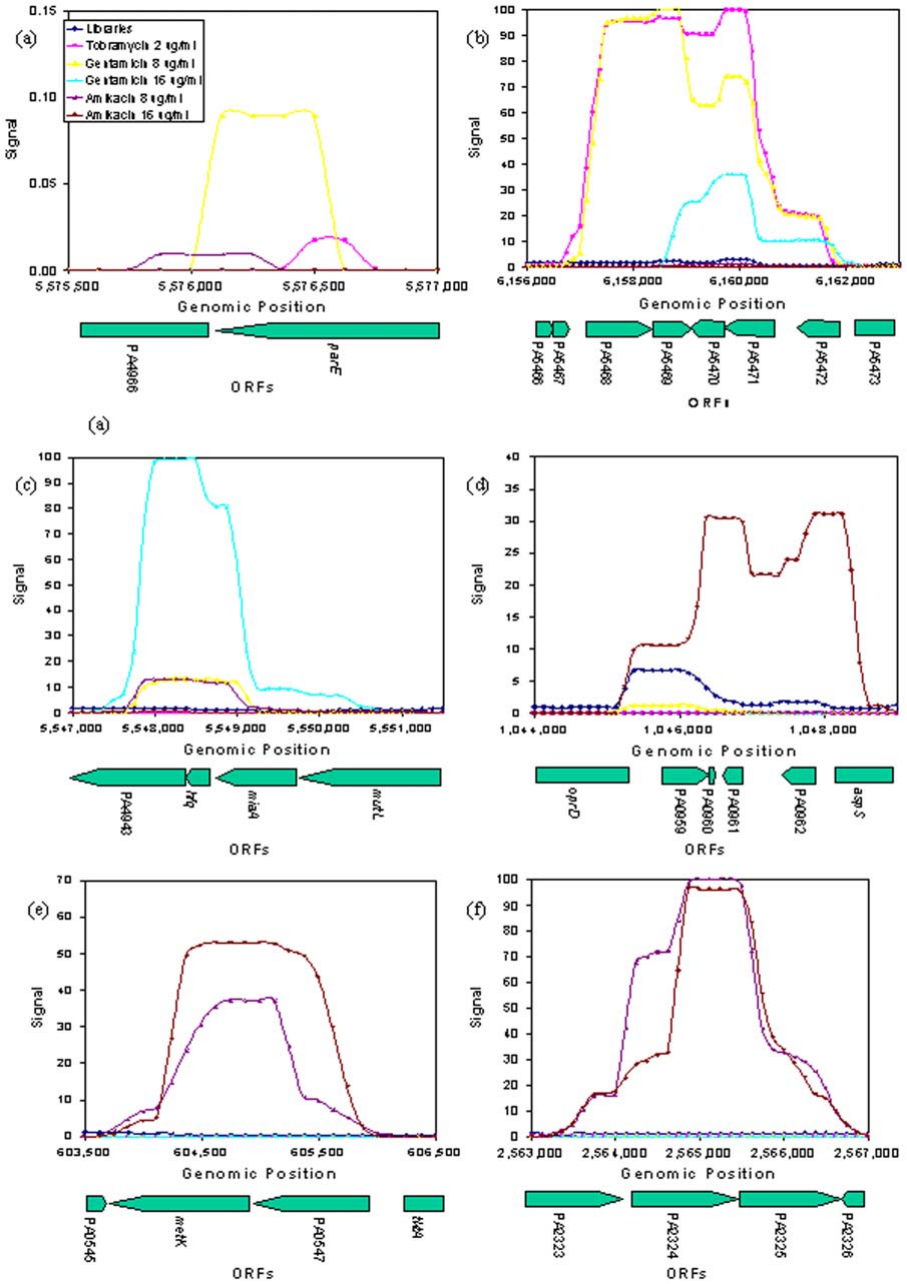
Contours of microarray signal intensity plotted according to genomic position for the genomic regions identified during sequencing, listed in **Table 1**. The regions highlighted are those of (A) clone T2-C1, (B) clone T2-C3, (C) clone G16-C1, (D) clone G16-C2, and the two regions contained within clone A8-C1 (E and F). The Y-axis on each individual graph depicts the relative microarray signal intensity. The X-axis depicts the genomic position in base pairs. Below the X-axis are the ORFs for which the depicted genomic region encompasses.

We next sought to assess enrichment patterns localized to specific genomic regions presented in Table 1. SCALEs employs multiple-sized libraries that can act cumulatively to give distinct contours to plotted signal intensity across genomic position that can be used to reveal genes or genomic regions that were enriched during a selection (Figure 3). Figures 3A through 3D depict the genomic regions found within clones T2-C1, T2-C9, G16-C1, G16-C2, respectively, while 3E and 3F depict the two insert regions found within clone A8-C1.

## Discussion

The overall objective of this work was to apply the SCALEs methodology to identify potential aminoglycoside resistance genes present in *P. aeruginosa*. Reaching this objective first required that we adapt the SCALEs methodology for use in *P. aeruginosa*, which could then be followed with studies focused on revealing resistance genes and assessing overlap in genome-scale enrichment patterns.

### Adaptation of SCALEs

The adaptation of SCALEs for use in *P. aeruginosa* proved challenging in several regards. First, genomic DNA extracted from *P. aeruginosa* PAO1 proved to be relatively fragile and thus limited the size of genomic libraries that could be created. Ideally, we would have included an 8 kb library in the selections so that larger genomic segments could be identified. Second, we chose to perform selections on agar plates to emphasize selection for survival instead of growth rate at increased aminoglycoside concentrations. Due to the limited number of clones that survived the selections, the clones harvested from the agar plates required a step that would increase the amount of plasmid DNA that could be extracted so that sufficient amount of genomic insert DNA would be available to detect a signal on a microarray. We chose to grow the cells planktonically in the presence of glucose to minimize the impact of the insert behind the pBAD promoter of the vector. This extra growth step needed to be taken into account while analyzing and making predictions about the microarray data. Third, as evidenced by the colonies that were capable of growing on higher aminoglycoside concentrations than the bulk within the control populations of *P. aeruginosa* PAO2003, mutation, recombination, or the presence of persister cells could be a non-negligible portion of the population. This affirmed the need to confirm that the genomic DNA inserts within the plasmid of the selected library clones were indeed responsible for the noted increase in resistance. Consideration of these issues, along with host adaptability, is essential for others seeking to extend this method to additional bacterial species.

### Interpretation of Microarray Data

Despite these difficulties, the adapted SCALEs method proved to be successful at identifying genomic regions for which increased copy number promotes aminoglycoside resistance. Our data indicated that reservoirs of resistance genes overlapped significantly across the three aminoglycosides evaluated here. This result suggests that at least in the earliest stages in the evolution of aminoglycoside resistance, treatment with one aminoglycoside has the potential to result in the selection for mechanisms effective against additional aminoglycosides. This interpretation was bolstered by our aminoglycoside resistance data (see Table 1) where each of the five clones evaluated showed cross-resistance.

Comparison of enrichment patterns across experiments (Figure 2) can provide some insight regarding the parallel evolution of resistance phenotypes. For example, cross resistance to gentamicin and tobramycin has been reported in strains containing particular aminoglycoside modifying enzymes [6], [33] that have not shown significant resistance to amikacin. Interestingly, the clustering algorithm grouped several loci with PAO0959-0963, including *potGH, moaCDE*, *rpmB* and *rpmG*, among others. Clones containing such genes were not found in our sequencing studies, demonstrating the utility of a microarray approach capable of identifying not only the clones present at highest concentrations (i.e. those in Table 1) but also those clones present at reduced concentrations that still may confer a selective advantage and be worthy of additional study (not all clones are present at equal concentrations in the starting population).

Given what is known about clinical resistance [25], we expected to find a larger number of genes encoding enzymatic functions that could potentially be acting to modify aminoglycosides or functions involved in cell membrane permeability. Surprisingly, the majority of the dominating regions included genes encoding transcriptional or translational functions.

The parE gene encoding a topoisomerase IV subunit B was identified via sequencing of a clone from the tobramycin selection and within the microarray data from the gentamicin and tobramycin selections. As shown in Table 1, the *parE* clone exhibited 2–16 fold increased resistance across all three aminoglycosides. Mutations within *parE* in *P. aeruginosa* [34], *E. coli*, *Salmonella*, [35] and other species [36], [37] have been shown to increase resistance levels to quinolones antibiotics. It is known that quinolones target two essential enzymes, DNA gyrase and topoisomerase IV [38], involved in DNA replication, repair, transcription, and recombination which allows for their bactericidal activity. Prior studies have linked the aminoglycoside antibiotics with interfering with the initiation of DNA replication [39], [40], but no studies that we know of have yet been published that show aminoglycoside antibiotics inhibiting known ParE function, the separation of copied daughter DNA molecules, which would explain how overexpression of *parE* could overcome this effect of aminoglycosides.

Several different regions confirmed to increase resistance contained genes encoding transcriptional and translational functions [*hfq* (host factor I), PA0961 (probable cold-shock protein), *aspS* (aspartyl-tRNA synthetase), and PA0547 (probable transcriptional regulator)]. Of particular note, both *hfq* [41], [42] and cold shock proteins [43] are known to be involved in the stress response and have been shown to have RNA chaperone functions allowing them to influence the stability of mRNA transcripts. Moreover, *hfq* has been implicated in small RNA regulation of mRNAs [42], [44], efficient translation of the sigma-factor encoding *rpoS* [45], [46], and, most recently, interference with mRNA binding to the 30S ribosomal subunit [47], the same ribosomal subunit to which aminoglycosides bind [23], [48], [49]. It is noteworthy that *hfq* was the only full ORF contained within our microarray data covering this region of the genome (Figure 3c).

These findings raised the question of whether increased copy number of these stress-related genes lead to increased resistance not only to aminoglycoside but to other classes of protein inhibitors and other non-ribosomal targeting antibiotics. Increased resistance to multiple classes of antibiotics would indicate that increased copy number of these genes lead to a more general stress effect on antibiotic resistance as opposed to an effect specific to aminoglycoside resistance. No increased resistance was noted (data not shown) for clones T2-C1, G16-C1, or G16-C2 to chloramphenicol ciprofloxacin, or tetracycline.

Though there were a large number of genes involved in transcriptional and translational processes enriched within the selected populations, as expected there was also a number of genes encoding permeability related functions. Recently, Morita *et al.* [50] have published that PA5471 is necessary for MexXY efflux expression and thus MexXY-mediated efflux pump aminoglycoside resistance. Furthermore, they reported that PA5470 (annotated as a probable peptide chain release factor) is not necessary for MexXY expression. This report coincides with our microarray data that consistently peaked over PA5471 relative to PA5470 (Figure 3b). This data in particular points out the utility of the SCALEs approach that relies upon the use of multiple libraries containing defined insert sizes and microarrays to efficiently evaluate the entire library population. It should be noted that while this genomic region was identified both by microarray and sequencing of an individually selected clone, the directionality of the genomic insert behind the pBAD promoter was revealed only with sequencing. Sequencing revealed that the first ORF behind the promoter was PA5471, leaving directionally opposite PA5468 and PA5469 dependent upon a different promoter for expression. This highlights a limitation of identifying genomic regions with SCALEs and bolsters the finding that PA5471 is the important ORF related to increased resistance. The genes MexX and MexY were not identified by this method. This is most likely due to the size of MexX and MexY together is greater than 4 kb, the largest size of genomic insert included in the selections.

Alterations in membrane permeability due to membrane features may have also been responsible for the increased tolerance of aminoglycosides to clones within the selected populations. Selections enriched genomic segments containing genes related to O-antigens including *wbpM, wbpJ, wbpI, wbpH, wbpG* (involved in O-antigen biosynthesis), *wzx, wzy, and wzz* (involved in O-antigen assembly). A definitive link between O-antigens and aminoglycoside resistance has yet to be established, though there have been studies supporting the idea of altered or increased amounts of O-antigen structures could increase resistance levels to aminoglycosides. The expression of O-antigen biosynthesis and assembly genes have been found to have a 2- to 4 log-fold decrease in expression between amikacin-sensitive mutants generated from an aminoglycoside-resistant clinical isolate of *P. aeruginosa* compared to the resistant parent isolate [30].

Recently, Kohanski, *et al*. [51] published findings that bactericidal activity of an antibiotic is due to induced hydroxyl radical damage, caused by NADH depletion and disruption of iron-sulfur clusters, releasing to Fe^2+^ which then induces the Fenton reaction. They reported that a decrease in NADH supply, resulting in a decrease of superoxide generation, resulted in increased survival to bactericidal antibiotics. Furthermore, they reported that bactericidal drugs lead to DNA and protein damage. This work suggested that genes that would mitigate DNA or protein damage caused by hydroxyl radicals as well as genes preventing the accumulation of NADH could be used to mitigate the effects of bactericidal antibiotics. Our microarray analysis revealed an enrichment of regions including a number of genes involved in DNA repair and replication (including genes encoding topoisomerase I and a DNA mismatch repair protein, *mutL*) as well as a region including the gene *ndh*, encoding a NADH dehydrogenase. An extended list of genes identified during each antibiotic selection is included in the Additional file.

In conclusion, we have performed a genome-wide identification of genomic regions in *P. aeruginosa* PAO1 that have the potential to function in the evolution of aminoglycoside resistance. Overall, we found a large number of genomic regions could be recruited to increase the ability of *P. aeruginosa* to survive under aminoglycoside stress. A significant amount of overlap was observed among discovered regions for which increased copy number leads to elevated resistance levels to the three aminoglycosides examined. We have isolated and confirmed the increase in resistance and cross-resistance associated with five of these identified regions. The prominent open reading frames identified are annotated to have regulatory, transcriptional, translation, or cell envelope biogenesis functions, or are classified as only having hypothetical functions. The genomic regions identified provide insight into the evolution of aminoglycoside antibiotic resistance genes and indirect insight into the mode of action of aminoglycosides by observing how a cell may become more tolerant of aminoglycoside-induced stress. The number of genomic regions identified that are directly or indirectly related to translation or post-translation processes bolster the theory that the bactericidal action of aminoglycosides is correlated with interference with protein synthesis. Although not the focus here, this work opens many future research opportunities to further study and characterize the genetic and mechanistic bases for the increased resistance phenotypes of clones containing the identified genomic regions. Increased study on these genomic regions will add to the information about how a clinically relevant species such as *P. aeruginosa* can employ changes within these genomic regions to develop resistance and thus aid in planning therapeutic strategies to circumvent the rise of resistance as well provide information as to whether given cellular mechanisms may be targeted for future novel antibiotic therapies. Furthermore, this study demonstrates how the method of SCALEs can be adapted to find genes underlying an interesting phenotype, such as resistance, but also highlights the limitations associated with this approach and limitations that are imposed by decisions made upon experimental setup and experimental system in regards to the SCALEs method.

## Methods

### Strains, Culturing Conditions, and Antibiotics

*P. aeruginosa* strains PAO1 [52] and PAO2003 (*arg*H32 *str*A39 *chl*-2 *rec*A2) [53] were obtained from the *Pseudomonas* Genetic Stock Center. Strains were cultured at 37°C at 225 rpm in either Luria-Bertani (LB) broth or EZ Rich Defined Medium (EZ RDM) [54] (Teknova, Inc., Hollister, CA) using 0.5% glycerol as a carbon source and supplemented with 0.2% arabinose [55] where indicated. Many standard rich mediums, such as Luria Bertani, have batch-to-batch variations [56] and may have variations within calcium and magnesium ion concentrations, both of which have been shown to antagonize the affect of aminoglycosides [57]. To circumvent these issues, we chose to use a defined medium that was still rich in amino acids and nutrients, and thus we chose to use EZ RDM. Plate studies were conducted on EZ RDM agar plates incubated at 37°C. Gentamicin sulfate, tobramycin sulfate (both Research Products International, Mount Prospect, IL) and amikacin (Fisher Scientific, Pittsburgh, PA) were added to agar plates or medium at different concentrations, ranging from 1 to 128 µg/ml. Carbenicillin was added where indicated at a concentration of 100 µg/ml.

### Construction of Genomic Libraries

Genomic DNA from an overnight culture of *P. aeruginosa* PAO1 grown in LB broth was extracted using Qiagen (Valencia, CA) 500/G Genomic-tips and Genomic DNA Buffer Set following the protocol of the supplier with the exception of increasing the amount of wash buffer used to 3x the recommended volume. Partial digestion of genomic DNA was conducted with *Rsa* I and *Hae* III (Invitrogen, Carlsbad, CA). For 1 kb and 2 kb fragments, 1 and 2 kb bands were extracted from a partial digestion of genomic DNA resolved on a 1% standard agarose (Sigma-Aldrich, St. Louis, MO) using a MinElute Gel Extraction Kit (Qiagen). For 4 kb-sized fragments, a partial digestion was resolved on an analytical grade low melting point agarose gel (Promega, Madison, WI) and 4 kb-sized fragments of DNA excised. DNA was recovered using a GELase™ Agarose Gel-Digesting Preparation (EPICENTRE Biotechnologies, Madison, WI).

All plasmid extractions performed throughout this work were prepped using either a QIAprep Spin Miniprep Kit or HiSpeed Plasmid Midi Kit (Qiagen). Vector pBTB-1 was linearized by digestion with *Hinc* II and dephosphorylated with Antarctic Phosphatase (both New England BioLabs, Ipswich, MA). T4 DNA Ligase (Lucigen) was used to ligate linearized vector that had not been successfully dephosphorylated. Linearized and ligated vector were separated on a 1% agarose gel. Linearized vector was excised from the gel and the DNA extracted using a MinElute Gel Extraction Kit (Qiagen).

Ligation reactions and transformations were carried out using an UltraClone™ DNA Ligation and Transformation Kit (Lucigen). To increase the number of transformants, prior to transformation, ligations were precipitated with yeast tRNA [58]. Yeast tRNA precipitations were performed by adding to a 10 µl ligation 2 µl of 1 µg/µl yeast tRNA (Invitrogen), 28 µl of ultrapure water, and 100 µl of ice-cold absolute ethanol and placing the mixture in a −20°C freezer for 15 minutes. The mixture was then centrifuged at 16,000×g for 15 minutes at 4°C. The pellet was washed once with 200 µl of 70% ethanol, air dried, and finally resuspended in 2 µl of ultrapure water. Over 100,000 transformants per library were generated with this method. Genomic libraries were prepared from pooled colonies harvested from transformation plates using a HiSpeed Plasmid MidiKit (Qiagen).

### *P. aeruginosa* Transformations

Electrocompetent *P. aeruginosa* PAO2003 were prepared using the method of Choi, *et al.* [59] in which, for each transformation, 6 mls of an overnight culture in LB medium was centrifuged at 16,000×g for 1 minute at room temperature, the pelleted cells washed twice with room temperature 300 mM sucrose, and cells resuspended to a total volume of 50 µl using room temperature 300 mM sucrose. These electrocompetent cells were mixed with genomic libraries, transferred into a Fisherbrand 1 mm gap electroporation cuvette (Fisher Scientific), and electroporated at 2.5 kV with a Eppendorf Electroporator 2510 (Eppendorf, Westbury, NY). Transformations were recovered in 1 ml of LB medium for 2 hours at 37°C and 225 rpm prior to plating onto prewarmed LB agar plates supplemented with 100 µg/ml carbenicillin. Transformants were scraped from the plates, and resuspended in EZ RDM supplemented with glycerol. Cryogenic freezer stocks were made with resuspended cells by combining equal volumes of cell suspension and a 30% glycerol solution.

### Sequencing and Annotation Information

Sequencing was performed by Macrogen (Seoul, Korea) using the primers (5′-CAG TCC AGT TAC GCT GGA GTC-3′) and (5′-TAT CGC AAC TCT CTA CTG TTT C-3′). All annotation information was taken from the *Pseudomonas* Genome Database [60].

### Selections

3×10^7^ cells from the freezer stock of the 1 kb, 2 kb, and 4 kb libraries were combined into to 10 ml of EZ RDM. The cells were allowed to recover for 1 hour at 37°C and 225 rpm. 3×10^6^ cells were plated onto each EZ RDM agar plate containing 0.2% arabinose and increasing concentrations of gentamicin, amikacin, or tobramycin. *P. aeruginosa* PAO2003 and *P. aeruginosa* PAO2003 harboring pBTB-1 were used as controls. The concentration of antibiotic that prevented growth was designated the minimum inhibitory concentration (MIC). Plates with growth from the library selection that had concentrations of antibiotic higher than the MICs of the controls were saved for further studies. From these plates, a number of colonies were individually picked, grown planktonically, and prepared for plasmid extraction. The recovered plasmids were then sequenced. Colonies remaining on the plates were scraped into EZ RDM and combined with an equal volume of 30% glycerol and saved as cryogenic freezer stock. Selection studies were performed in duplicate to ensure repeatability (with comparable number of surviving clones found for each selection).

### Preparation of Samples for Microarray Studies

In order to obtain sufficient amounts of plasmid DNA to ensure a detectable signal when hybridized to a microarray, 0.1 ml of freezer stock from each selection was inoculated into 200 ml of LB medium supplemented with carbenicillin and 0.4% glucose. These cultures were grown to an OD_600_ of approximately 1. Plasmid DNA was extracted with a HiSpeed Plasmid Midi Kit (Qiagen) and prepared for hybridization similarly to that described previously [1]. This entailed mixing 7.5 µg of sample plasmid with 1,000 ng of pGIBS-DAP (ATCC#87486), 100 ng of pGIBS-THR (ATCC#87484), 10 ng of pGIBS-TRP (ATCC#87485), and 1 ng of pGIBS-PHE (ATCC#87483) and digesting the mixture overnight with *Rsa* I and *Hae* III (Invitrogen). The heat-inactivated reaction was supplemented with 10X One Phor All buffer (Amersham Pharmacia Biotech, Piscataway, NJ) to a final concentration of 1X and plasmid DNA was further digested and made single stranded by the addition of 2 units of RQDNAse I (Fisher Scientific) and 200 units of Exonuclease III (Fisher Scientific) incubated at 37°C for 30 minutes, followed by heat inactivation at 98°C for 20 minutes. This single-stranded DNA was labeled with biotinylated ddUTP using and Enzo BioArray Terminal Labeling kit (Enzo Life Sciences, Farmingdale, NY) according to the instructions provided by the manufacturer. Target hybridization, washing, staining, and scanning were performed on *P. aeruginosa* Genome Arrays (Affymetrix, Santa Clara, CA) at the University of Colorado DNA Microarray Facility according to the manufacturer's specifications.

### Microarray Data Analysis

Low level probe analysis, noise reduction, and probe signal mapping to their positions along the *P. aeruginosa* PAO1 genome were performed with probe-level signals extracted from Affymetrix .cel files as detailed in Lynch, *et al.* [1]. The probe signal across the genome was broken down to segments of 125 base pairs. A histogram of the signal intensities for every 125 bp segment revealed the data has a large range with most segments having relatively minimal signal and a small number of segments having relatively large signal intensities. Removing the signal intensities from the top 500 segments on each chip, the mean and standard deviation of the signal intensities from the remaining segments were calculated. To focus upon the most dominant peaks, we removed segments that had signal intensities less than 4 standard deviations from the mean. The 500 segments displaying the highest signal intensities were then added back into the data. Microbial Genome Viewer was used to depict microarray data along genomic position [61]. Hierarchical clustering was conducted between arrays and probe signal mapped to genomic position and corresponding ORFs using complete linkage and a Spearman correlation. Microarray data is available through ArrayExpress (accession: E-MEXP-2396).

### Confirmation Studies

To ensure the noted changes in phenotype displayed by an isolated colony after a selection were due to the genomic DNA insert within pBTB-1, plasmid DNA was purified from individual clones from each selection and transformed into freshly prepared electrocompetent *P. aeruginosa* PAO2003. To confirm MICs of the individual clones, 10 µl of freezer stock of the retransformed clones was placed into 1 ml of EZ RDM and allowed to recover for 1 hour. 1 µl of this culture was then spotted upon EZ RDM agar plates containing 0.2% arabinose and increasing concentrations of amikacin, gentamicin, and tobramycin. These plates were allowed to incubate up to 60 hours and the concentrations of antibiotic that prevented growth noted.

## Supporting Information

Table S1 Extensive table of regions identified in each selection. Start and stop codons of regions are mentioned along with annotation of ORFs found within those regions.
(0.05 MB XLS)
